# Assessment of the cytolytic potential of a multivirus-targeted T cell therapy using a vital dye-based, flow cytometric assay

**DOI:** 10.3389/fimmu.2023.1299512

**Published:** 2023-12-22

**Authors:** Kiriakos Koukoulias, Penelope G. Papayanni, Julia Jones, Manik Kuvalekar, Ayumi Watanabe, Yovana Velazquez, Sarah Gilmore, Anastasia Papadopoulou, Ann M. Leen, Spyridoula Vasileiou

**Affiliations:** ^1^Center for Cell and Gene Therapy, Baylor College of Medicine, Texas Children’s Hospital and Houston Methodist Hospital, Houston, TX, United States; ^2^AlloVir, Waltham, MA, United States; ^3^Hematology Department- Hematopoietic Cell Transplantation Unit, Gene and Cell Therapy Center, “George Papanikolaou” Hospital, Thessaloniki, Greece

**Keywords:** viral infection, adoptive T cell immunotherapy, virus specific T cells (VSTs), potency assays, T cell cytotoxicity, chromium release assay, flow cytometric analysis, vital dye staining

## Abstract

Reliable and sensitive characterization assays are important determinants of the successful clinical translation of immunotherapies. For the assessment of cytolytic potential, the chromium 51 (^51^Cr) release assay has long been considered the gold standard for testing effector cells. However, attaining the approvals to access and use radioactive isotopes is becoming increasingly complex, while technical aspects [i.e. sensitivity, short (4-6 hours) assay duration] may lead to suboptimal performance. This has been the case with our ex vivo expanded, polyclonal (CD4+ and CD8+) multivirus-specific T cell (multiVST) lines, which recognize 5 difficult-to-treat viruses [Adenovirus (AdV), BK virus (BKV), cytomegalovirus (CMV), Epstein Barr virus (EBV), and human herpes virus 6 (HHV6)] and when administered to allogeneic hematopoietic stem cell (HCT) or solid organ transplant (SOT) recipients have been associated with clinical benefit. However, despite mediating potent antiviral effects *in vivo*, capturing *in vitro* cytotoxic potential has proven difficult in a traditional ^51^Cr release assay. Now, in addition to cytotoxicity surrogates, including CD107a and Granzyme B, we report on an alternative, vital dye -based, flow cytometric platform in which superior sensitivity and prolonged effector:target co-culture duration enabled the reliable detection of both CD4- and CD8-mediated *in vitro* cytolytic activity against viral targets without non-specific effects.

## Introduction

Severe or fatal infections with a broad array of viruses remain a frequent problem for immunocompromised individuals such as recipients of allogeneic hematopoietic stem cell (HCT) or solid organ transplants (SOT) (1, 2). Although treatment with small molecule anti-viral drugs may benefit some individuals, for many viruses they are of limited efficacy, have substantial toxicities and may generate resistant variants (1, 2). An alternative treatment strategy is to adoptively transfer T cells that are specific to viral antigens. Following HCT for example, administration of donor-derived T cells with specificity for viruses including cytomegalovirus (CMV), adenovirus (AdV), and/or Epstein Barr virus (EBV) have all produced frequent and sustained anti-viral and clinical benefits, even for patients suffering from advanced and drug-resistant infections (3, 4). More recently, “off the shelf”, or banked, partially HLA-matched virus-specific T cells (VSTs) have shown promise in treating intractable viral infections in SOT and HCT recipients (5–8).

To maximize patient benefit, our group developed an approach to generate a single preparation of VSTs containing a polyclonal (CD4+ and CD8+) mixture of T cells specific for immunodominant antigens from CMV, AdV, EBV, human herpesvirus 6 (HHV6), and BK virus (BKV) – all viruses that are frequently detected in the post-transplant period. The VSTs were expanded from the memory T cell pool within peripheral blood mononuclear cells (PBMCs) isolated from seropositive donors and were enriched and selectively expanded over a 2-week ex vivo culture period (9). When infused to patients in proof of concept clinical trials, either as individualized donor-specific (4) or “off the shelf” partially HLA-matched products (10) the VST infusions were safe and effective, resulting in virologic and clinical benefit to patients with drug-refractory infections and disease. Based on these promising results the efficacy of this product is now being explored in multiple, randomized, Phase III clinical trials (NCT05179057, NCT05305040, NCT04390113).

Prior to clinical use these multiVSTs are extensively characterized *in vitro* in order to assess the phenotype, potency/specificity, functional capacity and alloreactive potential of the expanded cells. Our cells are polyclonal (CD4+ and CD8+), selectively upregulate activation markers upon exposure to virus, and produce an array of Th1-polarized cytokines/effector molecules, consistent with effective and protective memory T cells. However, the cytolytic capacity of these memory T cells against viral targets cannot be reliably captured *in vitro* when assessed in a traditional chromium (^51^Cr) release assay – despite mediating potent antiviral effects in patients. Thus, the goal of the current study was to interrogate this *in vitro* phenomenon by exploring both cytotoxicity surrogates and novel cytotoxicity assay platforms to better capture and characterize the cytolytic potential of our effector VSTs.

## Materials and methods

### Donors and cell lines

Peripheral blood mononuclear cells (PBMCs) were obtained from healthy volunteers with informed consent using a Baylor College of Medicine institutional review board-approved protocol (H-45017) and were used to generate phytohemagglutinin (PHA) blasts and multiVSTs. PHA blasts were generated as previously reported (9) and cultured in T cell medium [45% RPMI 1640 (HyClone Laboratories, Logan, Utah), 45% Click’s medium (Irvine Scientific, Santa Ana, California), 2 mM GlutaMAX TM-I (Life Technologies, Grand Island, New York), and 5% human AB serum (Valley Biomedical, Winchester, Virginia)] supplemented with 100U/ml interleukin 2 [IL2; Proleukin^®^ (aldesleukin), TCH, Houston, TX], which was replenished every 2 days.

### Pepmixes

For multiVST generation and functional studies, pepmixes (15mers overlapping by 11aa) spanning AdV (Hexon, Penton), BKV (large T, VP1), CMV (IE1, pp65), EBV (EBNA1, BZLF1, LMP2), and HHV6 (U11, U14, U90) (JPT Peptide Technologies, Berlin, Germany) were synthesized. Lyophilized pepmixes were reconstituted in dimethyl sulfoxide (DMSO) (Sigma-Aldrich) and stored at -80°C.

### multiVST generation

To generate research-grade multiVSTs for characterization studies, PBMCs (1.25x10^7^) were transferred in a G-Rex5M (Wilson Wolf Manufacturing Corporation, St. Paul, MN) with 50 ml of VST medium [90% TexMACS™ GMP medium (Miltenyi Biotec, GmbH), 2 mM GlutaMAX, and 10% human AB serum] supplemented with IL7 (20 ng/ml), IL4 (800 U/ml) (R&D Systems, Minneapolis, MN) and pepmixes (2 ng/peptide/ml) and cultured for 14 ± 2 days at 37°C, 5% CO_2_ prior to cryopreservation. MultiVSTs were cryopreserved in freeze medium [45% RPMI + 45% Fetal Bovine Serum (FBS; Gibco) + 10% DMSO] at a concentration of 1.5-2x10^7^ cells/ml/cryovial. After overnight storage at -80°C in a Mr. Frosty freezing container (Thermo Fisher Scientific), cells were transferred to a liquid nitrogen tank for long-term storage in the vapor phase.

### Flow cytometry

#### Surface immunophenotyping

MultiVSTs were surface-stained with monoclonal antibodies to: CD45RO, CD197 (CCR7), CD279 (PD-1) [Becton Dickinson (BD), Franklin Lakes, NJ], CD3, CD4, CD8, CD16, CD56, CD62L (Beckman Coulter, Brea, CA), and CD366 (TIM-3), CD223 (LAG3) (BioLegend, San Diego, CA). Cells (0.5x10^6^ per tube) were pelleted in phosphate-buffered saline (PBS) (Sigma-Aldrich), then antibodies added in saturating amounts (5 μl) followed by incubation for 15 mins at 4°C. Subsequently, cells were washed, resuspended in 300μl of PBS and at least 50,000 live cells acquired on a Gallios™ Flow Cytometer (Beckman Coulter) and analyzed with Kaluza^®^ Flow Analysis Software (Beckman Coulter).

#### CD107a degranulation assay

MultiVSTs were harvested, resuspended in VST medium (2x10^6^/ml) and 200μl added per well of a round-bottom 96-well plate. Cells were incubated for 5-6 hrs with 200 ng of antigenic pepmixes pooled per virus along with CD107a, monensin (1 μg/ml), CD28 and CD49d (1 μg/ml) (BD). Next, multiVSTs were washed with PBS, pelleted, and surface-stained with CD3, CD4 and CD8 (5 μl/antibody/tube) for 15mins at 4°C. Subsequently, cells were washed, resuspended in 300 μl of PBS and at least 50,000 live cells acquired on a Gallios™ Flow Cytometer and analyzed with Kaluza^®^ Flow Analysis Software.

### Functional studies

#### Enzyme-linked immunospot

ELISpot analysis was used to quantitate the frequency of IFNγ- and Granzyme B-secreting cells. Briefly, multiVSTs were resuspended at 2x10^6^ cells/ml in VST medium and 100 μl of cells was added to each ELISpot well. Antigen-specific activity was measured after direct stimulation (500 ng/peptide/ml) with the antigenic pepmixes pooled per virus. After 16-18 hours of incubation, plates were developed as previously described (9), dried overnight at room temperature and then quantified using the IRIS ELISpot/FluoroSpot reader (Mabtech, Inc., Cincinnati, OH). Spot-forming cells (SFC) and input cell numbers were plotted. A threshold of ≥50 SFC/2x10^5^ input cells was used as the specificity threshold to confirm activity against individual target viruses but of note not all donors used in these experiments were seropositive for all target viruses.

### Cytotoxic potential

Two assays – (i) a chromium release assay and (ii) a vital dye cytotoxicity assay were used to evaluate cytolytic potential.

#### Chromium release assay

A standard 4-6 hour chromium (^51^Cr) release assay was used to measure the specific cytolytic activity of bulk multiVSTs or magnetically sorted CD4+ and CD8+ subsets (CD4 and CD8 microbeads and LS columns, Miltenyi Biotec) against autologous AdV, BKV, CMV, EBV, or HHV6 peptide-loaded PHA blasts as targets (20 ng/pepmix/1x10^6^ target cells). Effector : Target (E:T) ratios of 40:1, 20:1, 10:1, and 5:1 were used to analyze specific lysis. The percentage of specific isotope release was calculated as [(experimental release - spontaneous release)/(maximum release - spontaneous release)] x 100. In order to measure the autoreactive and alloreactive potential of VST lines, autologous and allogeneic PHA blasts alone were used as target cells.

#### Vital dye cytotoxicity assay

Cytotoxicity of bulk multiVSTs or magnetically sorted CD4+ and CD8+ T cell subsets against viral antigen-pulsed and unpulsed PHA blasts was measured with the carboxyfluorescein diacetate succinimidyl ester (CFSE) assay using a published protocol (11–14) with some optimization. In brief, unpulsed autologous PHA blasts were labeled with a high concentration of CFSE (CellTrace™ CFSE Cell Proliferation Kit; Invitrogen, Waltham, MA) (5 µM), while antigen-pulsed (20 ng/pepmix/1x10^6^ target cells) autologous or unpulsed allogeneic PHA blasts were labeled with a low concentration of CFSE (0.5 μM). A tumor-associated antigen (TAA)-derived pepmix (survivin, JPT Peptide Technologies) was used as irrelevant control. Both CFSE-labeled populations were mixed (1:1) and co-cultured with effector cells in a round-bottom 96-well plate at various E:T ratios; each PHA blast population was maintained at 2x10^4^ cells in all ratios. All conditions were plated in duplicates or triplicates. After 16 hrs of incubation, cells were stained with 7-AAD (7-aminoactinomycin D; BD) in order to exclude dead cells. A minimum of 5,000 viable CFSE-labeled target cells were acquired on a Gallios™ Flow Cytometer and analyzed with Kaluza^®^ Flow Analysis Software. In order to assess the reproducibility of the assay, an alternate fluorescent dye (PKH26 Red Fluorescent Cell Linker Midi Kit, Sigma-Aldrich) was used under the exact same experimental conditions. The percentage of specific cytotoxicity was calculated based on the ratio of CFSElow to CFSEhigh viable target cells in the test condition compared to the baseline (no effector cells): Cell lysis (%) = 100 – [100∗ Sample (CFSElow/CFSEhigh)/Baseline(CFSElow/CFSEhigh)].

## Results

### *In vitro* phenotypic and functional profiling of multiVSTs

**Figure 1:**
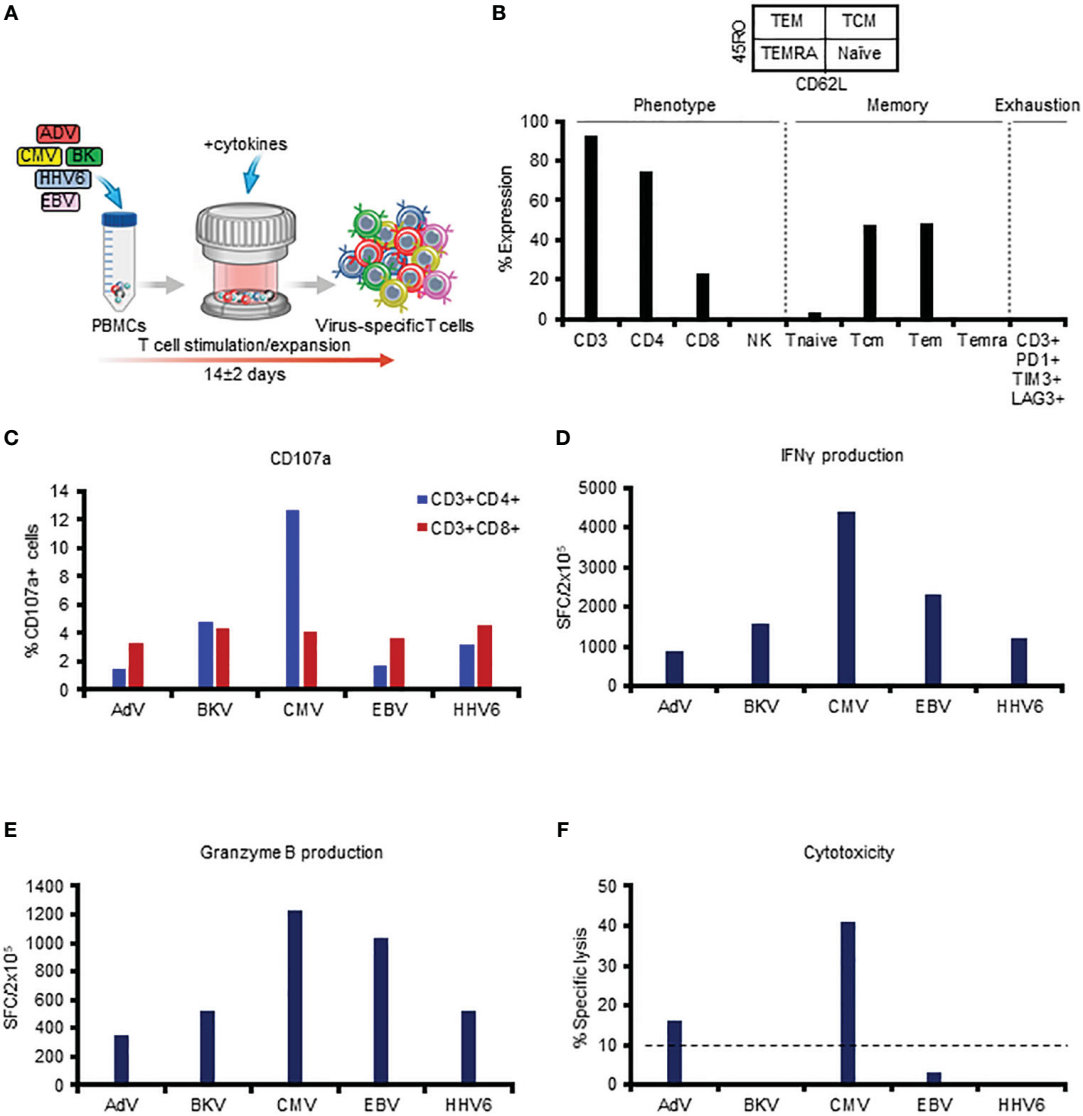
*In vitro* phenotyping and functional profiling of multiVSTs (representative donor). **(A)** Schematic representation of the manufacturing process of multiVSTs. **(B)** Surface phenotype of multiVSTs as assessed by flow cytometry. **(C)** Upregulation of CD107a by CD4+ and CD8+ multiVSTs following stimulation by individual target viruses. Unstimulated control values have been subtracted. **(D)** IFNγ production by multiVSTs as assessed by ELISpot assay using pooled pepmixes of each individual target virus as a stimulus. Results are expressed as SFC/2x10^5^ input cells; a threshold of 50 SFC/2x10^5^ was set to define specificity. Unstimulated control values have been subtracted. **(E)** Granzyme B production by multiVSTs as assessed by ELISpot assay using pooled pepmixes of each individual target virus as a stimulus. Results are expressed as SFC/2x10^5^ input cells. Unstimulated control values have been subtracted. **(F)** Specific cytotoxic activity of multiVSTs against viral antigen-loaded autologous PHA blasts as evaluated in a standard 5-hour ^51^Cr release assay at 40:1 E:T ratio. The threshold for specific lytic activity against each individual target virus (indicated by the dashed line) was set at 10%.

The multiVST manufacturing process entails exposing PBMCs to overlapping peptide libraries spanning 12 immunodominant antigens from our target viruses (Hexon, Penton - AdV, Large T, VP1 - BKV, pp65, IE1 - CMV, EBNA1, LMP2, BZLF1 - EBV, and U11, U14, U90 - HHV6), followed by expansion for approximately 2 weeks in a G-Rex device (**Figure 1A**). **Figure 1** includes representative data illustrating the features of a typical multiVST line, which are similar to our previously published phenotypic data (4, 9, 10). Briefly, the multiVSTs are comprised almost exclusively of CD3+ T cells (92.4%), that are polyclonal (CD8+ - 23%; CD4+ - 74.9%) and express markers consistent with central and effector memory potential (CD45RO+/CD62L+: 47.4%; CD45RO+/CD62L-: 48.3%), with negligible naïve/terminally differentiated/exhausted populations (**Figure 1B** and **Supplementary Figure 1**). These cells are potent based on both phenotypic and functional characteristics following viral antigen exposure. For example, they upregulate the degranulation marker CD107a (**Figure 1C**), and produce Th1-polarized effector cytokines and molecules (IFNγ and Granzyme B, respectively), as shown in **Figures 1D, E**. Importantly, use of these sensitive detection methods enables assessment of not just multivirus specificity (i.e. functional activity when VSTs are exposed to a mastermix of the target antigens) but also specificity on the individual target virus level as shown in **Figures 1D, E** (IFNγ SFC/2x10^5^ - AdV:881; BKV:1577; CMV:4383; EBV:2300; HHV6:1189; Granzyme B SFC/2x10^5^ - AdV:348; BKV:520; CMV:1232; EBV:1034; HHV6:524). Thus, multiVSTs have functional activity for each of the target viruses, and based on this testing (and the fact that these cells are derived from the memory pool of individuals with protective immunity) we can infer cytotoxic capacity by measuring surrogates of lytic potential such as CD107a and Granzyme B. However, demonstrating evidence of direct cytolytic activity *in vitro* has proven challenging using a standard 4-6 hour ^51^Cr release assay. For example, the multiVSTs from our representative donor show lytic activity (defined as >10% specific lysis at E:T 40:1) against AdV (16% specific lysis) and CMV (41%) but not against BK (0%), EBV (3%) and HHV6 (0%) (**Figure 1F**), despite evidence that the line includes CD8 and CD4 T cells that are reactive against these 3 viruses, secrete Granzyme B and upregulate the degranulation marker CD107a upon antigen recognition. **Supplementary Figure 2** shows similar results for 4 other lines in which all characterization assessments indicate potency against the target viruses, with the exception of the ^51^Cr release assay.

### Investigating an additional cytotoxicity assay for *in vitro* characterization of multiVSTs

We sought to further explore the basis for the low measurable killing activity of multiVSTs against all target viruses *in vitro*. These virus-specific T cells are expanded from the polyclonal memory pool of healthy donors and as such are representative of endogenous (and protective) T cell immunity against each of the target viruses. Hence, we surmised that it was unlikely that these cells were physiologically impaired but rather that our findings reflected an *in vitro* phenomenon related to the sensitivity of the standard ^51^Cr release assay and/or the characteristics of our VST products, which often have higher CD4+ versus CD8+ T cell content. To investigate whether this was indeed the case we first explored the utility of a potentially more sensitive, longer-term *in vitro* killing assay based on the use of vital dyes for target labeling and flow cytometry as an assay readout.

**Figure 2:**
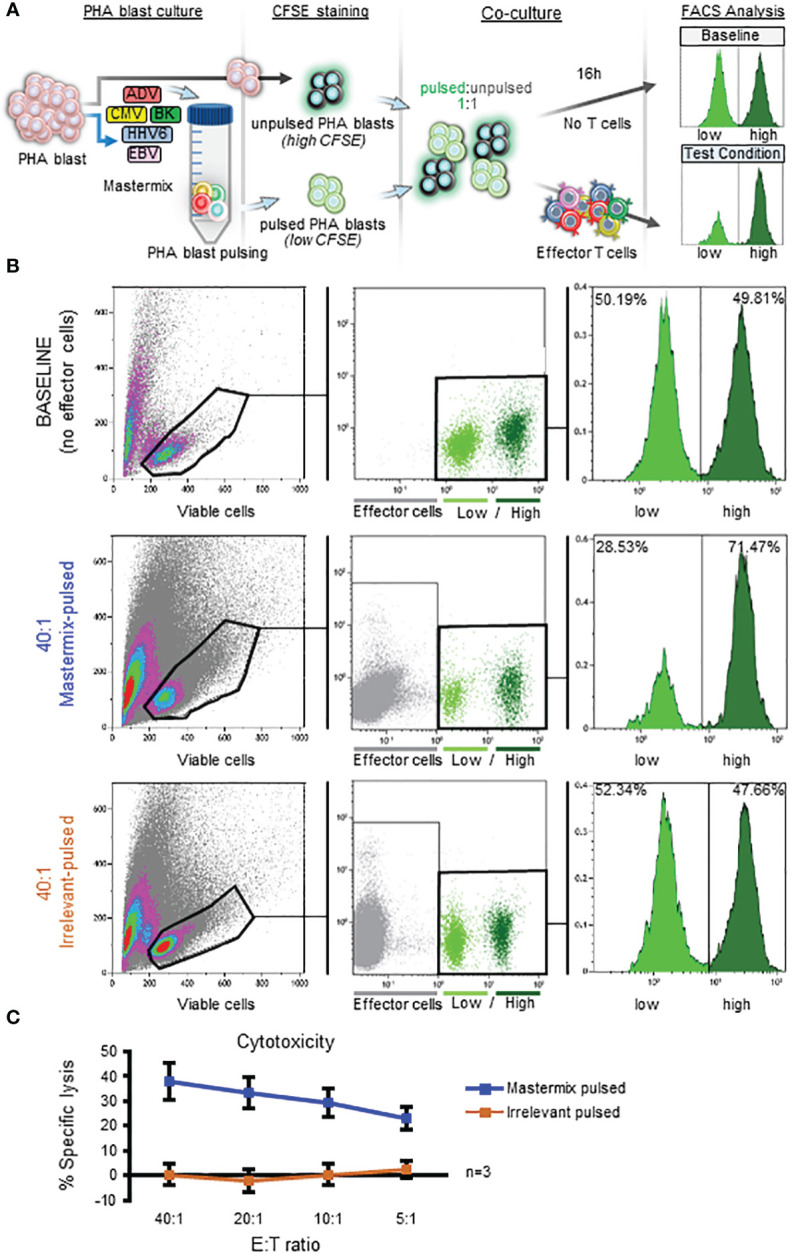
Implementation of a CFSE-based flow cytometric cytotoxicity assay for *in vitro* characterization of multiVSTs. **(A)** Schematic representation of the CFSE-based assay for assessment of specific cytolytic activity of effector T cells. Target cells (autologous PHA blasts) are either left unpulsed and labeled with a high (5 µM) CFSE concentration or are antigen-pulsed for 2 hours and labeled with a low (0.5 µM) CFSE concentration. Both CFSE-labeled populations are mixed 1:1 and co-cultured with effector T cells for 16 hours. Following addition of 7-AAD, specific lysis is evaluated by flow cytometry based on the ratio of viable ‘‘low’’ vs ‘‘high’’ targets in the test condition (antigen-loaded targets + T cells; bottom right histogram) compared to the baseline (no T cells; top right histogram). **(B)** Utilization of the CFSE-based assay to test the cytotoxic capacity of multiVSTs against autologous PHA blasts loaded with a mastermix of target viruses (AdV, BKV, CMV, EBV, HHV6); a representative donor is shown. Top panel – baseline condition (no VSTs). Middle panel – test condition (multiVSTs + mastermix-pulsed targets at 40:1 E:T). Bottom panel – control condition (multiVSTs + irrelevant peptide-pulsed targets at 40:1 E:T). **(C)** Cytolytic activity of multiVSTs against mastermix-pulsed (blue line) or irrelevant-pulsed (orange line) targets across several E:T ratios as assessed by the CFSE-based assay. Results are shown as mean ± SD (n=3).

For our initial studies target cells were labeled with CFSE, a non-fluorescent vital dye that passively diffuses across the cell membrane and subsequently undergoes esterase cleavage to render cells fluorescent (12, 15, 16). Thus, when fluorescently-labeled target cells are loaded with antigen and co-cultured with effector cells, elimination of fluorescent target cells serves as an indicator of specific lysis that can be evaluated by flow cytometry. In addition, and unlike a traditional ^51^Cr release assay in which ^51^Cr itself can induce toxicity or spontaneously diffuse from labeled cells following prolonged (i.e. overnight) incubation (17–19), effector and target co-culture can be extended beyond 6 hours without an increase in non-specific (background) lysis - an important feature given that the kinetics of CD4+ T cell killing are typically slower than those of CD8+ effectors (18–25). **Figure 2A** shows a schematic representation of the assay design in which target cells (autologous PHA blasts) are first loaded with cognate antigen (or left unpulsed) for 2 hrs. Antigen-expressing and non-expressing targets are then labeled with “low” (0.5 µM) or “high” (5 µM) CFSE concentrations, respectively, mixed 1:1 and co-cultured with effector T cells overnight. Specific lysis is calculated based on the ratio of residual ‘‘low’’ vs ‘‘high’’ targets (detected by flow cytometry) in the test condition (antigen-loaded targets + T cells; bottom right histogram, **Figure 2A**) compared to the baseline (no T cells; top right histogram, **Figure 2A**).

To first explore the feasibility of utilizing this CFSE-based cytotoxicity assay to assess the cytolytic potential of multiVSTs we pooled all target antigens. Briefly, as shown in **Figure 2B** (representative donor), in the absence of effector cells (baseline – top panel) the ratio of CFSE ‘‘low’’ to ‘‘high’’ targets remained unchanged over time. However, in the presence of effector multiVSTs, antigen-loaded targets were selectively lysed (40:1 mastermix-pulsed - middle panel). Importantly, co-culture of effector cells with irrelevant peptide-pulsed autologous targets did not induce specific lysis (40:1 irrelevant-pulsed – bottom panel). These results were reproduced in 2 additional donors across E:T ratios ranging from 40:1 to 5:1, as summarized in **Figure 2C**.

### Optimizing the CFSE platform for assessing cytotoxicity of multiVSTs

**Figure 3:**
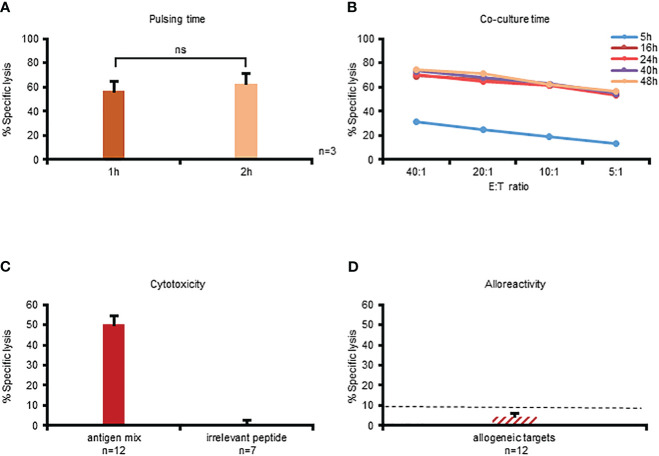
Optimization of the CFSE platform for assessing cytotoxicity of multiVSTs. **(A)** Impact of target antigen-pulsing time (1 hour vs 2 hours) on the killing activity of multiVSTs at 40:1 E:T following a 16-hour co-culture; results are shown as mean ± SD (n=3). **(B)** Impact of co-culture duration (5–48 hours) on detection of specific lysis across several E:T ratios; a representative donor is shown. C: Optimal detection of multiVST-mediated lysis of cognate antigen-pulsed targets (n=12) with no non-specific activity against irrelevant antigen-pulsed targets (n=7); results are shown as mean ± SEM. D: MultiVSTs exhibit minimal activity against allogeneic targets (n=12; results are shown as mean ± SEM). **(A-C)** autologous PHA blasts loaded with a mastermix of target viruses (AdV, BKV, CMV, EBV, HHV6) served as cognate antigen-pulsed targets. **(D)** HLA-mismatched PHA blasts served as allogeneic targets. **(C, D)** cytotoxicity was evaluated at 40:1 E:T using the optimized conditions, i.e. following a 1-hour target antigen-pulsing and a 16-hour E:T co-culture. The dashed line represents the threshold for specific lytic activity (>10%). Differences between data sets were analyzed using a 2-tailed Student’s t-test. P-values <0.05 were considered significant. ns, non-significant.

Having demonstrated the feasibility of utilizing the CFSE-based cytotoxicity assay we sought to optimize the assay conditions for VSTs by investigating 2 time-related parameters: (i) target antigen-loading time and (ii) E:T co-culture time. Traditionally the CFSE cytotoxicity assay is performed using targets that have been loaded with antigen for 2 hours (11, 13, 14) but we explored whether antigen loading for 1 hour would impact killing activity. As shown in **Figure 3A** there was no significant difference in effector T cell recognition (n=3). We next performed a time course experiment ranging from 5 hours (as used in the ^51^Cr release assay) to 48 hours to evaluate the impact of co-culture duration on specific lysis detection. As shown in **Figure 3B**, specific lysis was detected after 5 hours, but at levels that were substantially lower than all other timepoints (and in fact comparable to those detected in the standard ^51^Cr release; **Supplementary Figure 3A**). By extending the co-culture time to 16 hours the magnitude of specific lysis detected was substantially increased (**Supplementary Figure 3B**) but further gains were not achieved by prolonging incubation times (i.e. the specific lysis was maximally detected at 16 hours; **Figure 3B**). Based on this optimization work subsequent experiments were performed by loading targets with antigen for 1 hour and performing a 16-hour E:T co-culture; results of cytotoxicity assays from n=12 donors using these parameters are summarized in **Figure 3C**. Importantly, even with the extension of co-culture times, multiVSTs did not have non-specific activity against control (irrelevant antigen; **Figure 3C**) or allogeneic (HLA-mismatched; **Figure 3D**) targets.

**Figure 4:**
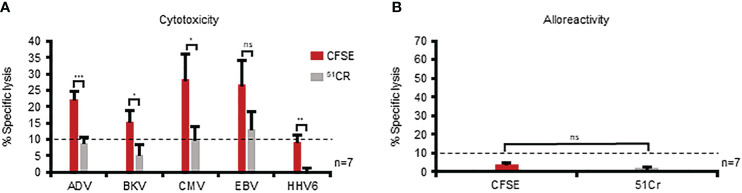
The CFSE-based assay is superior to standard ^51^Cr release in the detection of specific cytolytic activity of multiVSTs. **(A)** Assessment of the lytic capacity of multiVSTs against autologous PHA blasts loaded with individual target viruses using either a standard 5-hour ^51^Cr release assay (grey bars) or the 16-hour CFSE-based assay (red bars). **(B)** Lack of multiVST-mediated alloreactivity against HLA-mismatched PHA blasts as assessed by either a 5-hour ^51^Cr release assay (grey bar) or the 16-hour CFSE-based assay (red bar). Results are shown as mean ± SEM (n=7). Cytotoxicity was evaluated at 40:1 E:T. Dashed lines represent the threshold for specific lytic activity (>10%). %). Differences between data sets were analyzed using a 2-tailed Student’s t-test. P-values <0.05 were considered significant. ns, non-significant; *p=0.047; **p=0.002; ***p= 0.0004.

With an optimal set of parameters in place, we evaluated whether implementation of this CFSE-based assay would enable assessment of cytolytic activity against the individual target viruses recognized by multiVSTs. To that end, we pulsed autologous PHA blasts with AdV, BKV, CMV, EBV, or HHV6 antigens, labeled them with CFSE (or ^51^Cr as a comparator condition) and evaluated specific lysis at 40:1 E:T. As expected, the results from the 5-hour ^51^Cr release assay were heterogeneous and varied from donor-to-donor, with detection of specific lysis against 0/5 to 3/5 target viruses [mean ± sem: AdV: 8.9% ± 1.9, BKV: 5.4% ± 3.1, CMV: 9.9% ± 4.1, EBV: 13.2% ± 5.3, HHV6: 0.3% ± 0.9; n=7 (**Figure 4A**); individual donors are shown in **Supplementary Figure 4**]. In contrast, using the CFSE assay we increased the sensitivity of detection, enabling the measurement of specific lysis for at least 3 to all 5 of the target viruses (AdV: 22.3% ± 2.3, BKV: 15.4% ± 3.4, CMV: 28.4% ± 7.6, EBV: 26.7% ± 7.4, HHV6: 9.3% ± 2.1; n=7). Importantly, both the CFSE and ^51^Cr assays demonstrated no non-specific killing of allogeneic targets (**Figure 4B**). Taken together, these data demonstrate the superiority of the CFSE-based assay in the detection of specific cytolytic activity of multiVSTs compared to standard ^51^Cr release.

### CFSE-based measurement of cytotoxicity allows for successful detection of CD4-mediated specific killing

**Figure 5:**
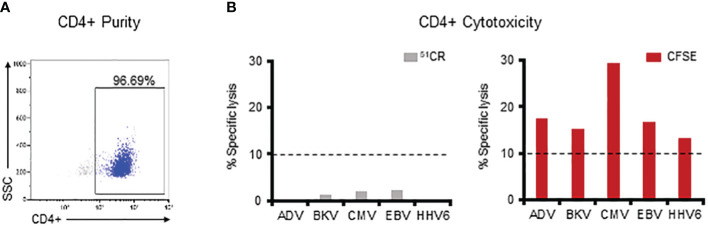
The CFSE-based assay allows for successful detection of CD4 T cell-mediated specific killing. **(A)** Purity of the magnetically sorted CD4+ VST fraction as assessed by flow cytometry. **(B)** Assessment of the lytic capacity of CD4+ multiVSTs against autologous PHA blasts loaded with individual target viruses using either a standard 5-hour ^51^Cr release assay (grey bars – left panel) or the 16-hour CFSE-based assay (red bars – right panel). A representative donor is shown. Cytotoxicity was evaluated at 40:1 E:T. Dashed lines represent the threshold for specific lytic activity (>10%).

Thus far we have established that multiVSTs exhibit cytolytic potential and can kill virus-expressing autologous targets but that such killing is not always detectable using the traditional ^51^Cr release assay. We posited that the high CD4+ T cell content of our multiVSTs, which receive just a single ex vivo stimulation, might contribute to lack of compatibility with the ^51^Cr release cytotoxicity assay. To specifically address this question, we magnetically sorted CD4+ T cells (**Figure 5A**) and evaluated their cytotoxic potential utilizing both the ^51^Cr release and CFSE cytotoxicity assay.

As shown in **Figure 5B** (left panel), we were unable to demonstrate CD4-mediated specific lysis against any of the viruses in the 5-hour ^51^Cr release assay. In contrast, by using the CFSE assay such cytolytic potential was readily measurable for CD4+ T cells (AdV: 17.4%, BKV: 15.4%, CMV: 29.3%, EBV: 16.7%, HHV6: 13.4%; **Figure 5B**-right panel). Similar results were obtained in 3 additional donors in whom CD4-mediated killing against each of the target viruses was consistently detected with the CFSE assay (**Supplementary Figure 5**), thus illustrating the requirement for prolonged E:T co-culture time and sensitive assessment methods (i.e. flow cytometry) (26–30) when seeking to measure CD4-mediated cytotoxic effects.

### The CFSE-based assay is robust and compatible with high-throughput flow cytometric platforms

**Figure 6:**
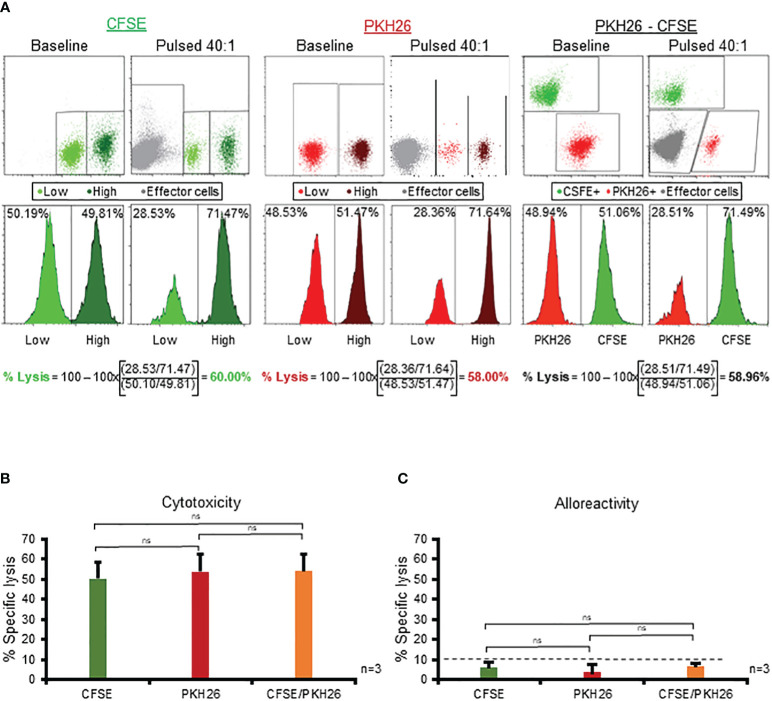
The CFSE-based assay is robust and compatible with high-throughput flow cytometric platforms. **(A)** Use of PKH26 (middle panel) or combination of PKH26 and CFSE (right panel) to differentially label antigen pulsed/unpulsed target cells yields identical results to CFSE (left panel). A representative donor is shown. **(B)** Comparable magnitude of specific cytotoxicity of multiVSTs against single- (either CFSE or PKH26) or dual- (CFSE/PKH26) stained target cells (n=3; mean± SD). **(C)** MultiVSTs exhibit minimal activity against single- (either CFSE or PKH26) or dual- (CFSE/PKH26) stained allogeneic targets (n=3; mean± SD). **(A, B)** autologous PHA blasts loaded with a mastermix of target viruses (AdV, BKV, CMV, EBV, HHV6) served as cognate antigen-pulsed targets. **(C)** HLA-mismatched PHA blasts served as allogeneic targets. Cytotoxicity was evaluated at 40:1 E:T following a 1-hour target antigen-pulsing and a 16-hour E:T co-culture. The dashed line represents the threshold for specific lytic activity (>10%). Differences between data sets were analyzed using a 2-tailed Student’s t-test. P-values <0.05 were considered significant. ns: non-significant.

Having demonstrated the superior sensitivity and high specificity of the CFSE-based assay in assessing specific cytolytic activity of multiVSTs, we wanted to examine the broad applicability of this platform by evaluating compatibility with: (i) fluorescent compounds beyond CFSE and (ii) different instruments. Thus we examined PKH26, a yellow-orange fluorescent dye that binds to lipid regions of the cell membrane and is commonly utilized in cell tracking applications due to its extremely stable fluorescence (16). Similar to our CFSE experimental design we used low (0.3 µμ) and high (3 µμ) concentrations of PKH26 to differentially label target cells, which could be readily distinguished from one another (and from effector T cells) by flow cytometry (**Figure 6A** - middle panel). The PKH26 and CFSE dyes could also be combined to discriminate antigen-loaded and unloaded targets (**Figure 6A** - right panel). Importantly, specific cytotoxicity was successfully measured using the alternate dye/combination of dyes, yielding almost identical results to CFSE (**Figure 6A** - left panel) in all cases (CFSE vs PKH26 vs PKH26/CFSE: 59.84% vs 58% vs 59% at 40:1 E:T; **Figure 6A**). **Figure 6B** shows summary results from 3 donors in whom we tested single (either CFSE or PKH26) or dual (CFSE/PKH26) staining of target cells; in all cases cytotoxicity readouts were similar among the different conditions with minimal non-specific cytolytic activity (**Figure 6C**).

Finally, we evaluated the compatibility of this vital dye cytotoxicity assay with different instrumentation by comparing the Gallios flow cytometer (an ultra-sensitive, high-speed 4-laser/10 fluorescent color detection system) to the CytoFlex, which is a novel 6-laser/21-color flow cytometry platform with the added potential for high-throughput data acquisition that can be performed in regulated environments (21 CFR part 11-compliant software). Results from a representative donor are shown in **Supplementary Figure 6A**; as demonstrated, CFSE low/high-labeled target cells were equally well separated and specific killing reliably assessed using either instrument. We repeated this testing in additional donors and reproducibly observed comparable magnitude of specific lysis measured by either system (**Supplementary Figure 6B**). Of note, the BD Accuri C6 flow cytometer also yielded comparable data (data not shown). Taken together, these results demonstrate the robustness of this vital dye platform, which yields reliable and reproducible measurements using variable fluorescent reagents and/or instrumentation. Notwithstanding the optimization work that may potentially be required (e.g. adjustment of experimental parameters depending on target cell type, assay validation for use in the clinical arena), this vital dye-based system can be broadly applicable in both research and clinical settings.

## Discussion

In this study, we explored novel strategies to evaluate the cytolytic potential of effector T cells *in vitro*. Having demonstrated that a standard ^51^Cr release assay consistently fails to effectively measure virus-directed lysis of our potent VSTs we subsequently explored an optimized, flow cytometry vital dye-based system in which prolonged E:T co-culture times and superior (flow cytometry-based) sensitivity enabled the detection of *in vitro* cytotoxic activity against viral targets without non-specific effects. Finally, we demonstrate the broad utility of this vital dye-based system, showing compatibility with different dyes and data capture on multiple flow cytometry instruments that are suitable for both pre-clinical and clinical applications. Though the current work focused on non-gene-modified, virus-specific T cells, the same platform could be applied to test the cytotoxic activity of tumor-specific T cells or tumor-infiltrating lymphocytes (TILs), as well as genetically modified CAR- or TCR-modified T cells.

Our multiVST cell lines, which are generated from healthy seropositive individuals with a single *in vitro* PBMC stimulation using overlapping peptide libraries spanning immunogenic target antigens, yield polyclonal T cells which contain both CD8+ and CD4+ T cell subsets. Thus, our multiVSTs are enriched for virus-specific T cells that reflect the diverse native (memory) T cell repertoire responsible for protective endogenous immune effects in healthy individuals. To characterize multiVST populations we use an array of techniques to evaluate the memory, activation, Th1-polarization, and polyfunctionality of the induced cells. To evaluate cytolytic capacity, surrogates of cytotoxicity such as upregulation of the degranulation marker CD107a and production of Granzyme B (31, 32), or direct target lysis can be assessed. However, the latter killing capacity of multiVSTs has proven difficult to measure in a traditional *in vitro* 4-6 hour ^51^Cr release assay, even though upon infusion to patients these cells have consistently demonstrated potent and direct antiviral effects. We hypothesized that this discrepancy was an *in vitro* (rather than a physiologic) phenomenon and likely related to the high CD4+ T cell content of our VSTs resulting in delayed killing kinetics that can take up to 72 hours – a timeframe that is incompatible with the traditional ^51^Cr release assay (18–20, 23, 24). Implementation of the flow cytometric vital dye-based assay, with its enhanced sensitivity and its amenability to prolonged (16 hours) E:T co-culture time, enabled the reliable detection of cytolytic activity of multiVSTs against each of the target viruses. To examine whether this was indeed due to the ability to measure CD4 T cell-mediated killing, we repeated our cytotoxicity testing on sorted CD4+ populations and confirmed that the CFSE-based platform (with its prolonged co-culture time) consistently enabled the measurement of *in vitro* cytolytic capacity against each target virus, whereas ^51^Cr release permitted only partial detection of CD4-driven cytotoxicity. Of note, the optimal E:T co-culture timeframe was 16-24 hours, and extending further did not result in enhanced detection.

The pivotal role that CD4+ T cells play in coordinating the different arms of the immune system - from providing B cell help (via cell-cell contact and cytokine support) to promoting effector CD8+ T cell responses and maintaining a functional memory CD8+ T cell pool via cellular interactions and cytokine production - has long been appreciated (33, 34). However, in recent years it has become increasingly clear that the effector functions of CD4+ T cells extend well beyond their “helper” role (35–37). For example, in SOT recipients infected with CMV, early induction of cytolytic CD4+ T cells (preceding CD8+ T cell responses) has been associated with asymptomatic disease (38), and their maintenance during latency has been correlated with viral control (39, 40). Similarly, in the setting of acute viral infections [e.g. influenza (41), hepatitis C (HCV) (42) and Hantaan virus (HTNV; the cause of hemorrhagic fever with renal syndrome) (43)], the presence of cytotoxic CD4+ T cells has been linked to viral clearance and milder disease. Indeed, HIV-specific cytolytic CD4+ T cell responses during acute infection are highly predictive of slow disease progression (44) and efficient control of viremia in chronically infected patients (45). Cytolytic CD4+ T cells have also been demonstrated to mediate potent anti-tumor effects [Hunder et al., 2008, Tran et al., 2014, Johnson et al., 2016, Rodig et al., 2018, Alspach et al., 2019, Tang et al., 2021 (46–51)]. For example, naturally occurring Granzyme A/B- and perforin-expressing CD4+ T cells with the ability to eliminate autologous tumor have been identified in multiple myeloma patients; higher frequencies of these cells were associated with lower numbers of circulating plasma cells and less advanced disease, thus indicative of better prognosis (52). More recently, Oh et al (53) identified multiple cytotoxic CD4+ T cell subsets within human bladder tumors that killed autologous tumors in an HLA class II-restricted manner and whose presence was predictive of clinical response to anti-PD-L1 in those with metastatic disease. Cachot et al (54) extensively described CD4+ T cell clusters across different human cancers (melanoma, breast cancer, head and neck cancer, and hepatocellular carcinoma) that displayed cytotoxic properties with direct, contact- and granzyme-dependent lytic activity against autologous tumors.

Thus, it is clear that both CD8+ and CD4+ T cells are capable of mediating polyfunctional and potent antigen-specific effects *in vivo* – not all of which can easily be assessed *in vitro*. While the complexities of measuring the direct cytolytic potential of such polyclonal, memory T cells were addressed in the current study, surrogate markers of functional capacity (e.g. CD107a and Granzyme B) should also be considered as measures that predict cytolytic potential of effector cells destined for clinical use.

## Data availability statement

The original contributions presented in the study are included in the article/**Supplementary Material**. Further inquiries can be directed to the corresponding author.

## Ethics statement

The studies involving humans were approved by Baylor College of Medicine institutional review board. The studies were conducted in accordance with the local legislation and institutional requirements. The participants provided their written informed consent to participate in this study.

## Author contributions

KK: Conceptualization, Investigation, Methodology, Software, Visualization, Writing – original draft. PP: Conceptualization, Investigation, Methodology, Visualization, Writing – original draft. JJ: Investigation, Software, Visualization, Writing – review & editing. MK: Investigation, Writing – review & editing. AW: Investigation, Writing – review & editing. YV: Investigation, Writing – review & editing. SG: Writing – review & editing. AP: Methodology, Writing – review & editing. AL: Conceptualization, Data curation, Funding acquisition, Supervision, Writing – original draft, Writing – review & editing. SV: Conceptualization, Data curation, Investigation, Supervision, Writing – original draft, Writing – review & editing.

## References

[1] Hill JA Mayer BT Xie H Leisenring WM Huang ML Stevens-Ayers T. The cumulative burden of double-stranded DNA virus detection after allogeneic HCT is associated with increased mortality. Blood (2017) 129:2316–25. doi: 10.1182/blood-2016-10-748426 PMC5399484 28209721

[2] Hill JA Moon SH Chandak A Zhang Z Boeckh M Maziarz RT. Clinical and economic burden of multiple double-stranded DNA viral infections after allogeneic hematopoietic cell transplantation. Transplant Cell Ther (2022) 28:619 e1–8. doi: 10.1016/j.jtct.2022.06.016 35764288

[3] Gerdemann U Katari UL Papadopoulou A Keirnan JM Craddock JA Liu H. Safety and clinical efficacy of rapidly-generated trivirus-directed T cells as treatment for adenovirus, EBV, and CMV infections after allogeneic hematopoietic stem cell transplant. Mol Ther (2013) 21:2113–21. doi: 10.1038/mt.2013.151 PMC3831033 23783429

[4] Papadopoulou A Gerdemann U Katari UL Tzannou I Liu H Martinez C. Activity of broad-spectrum T cells as treatment for AdV, EBV, CMV, BKV, and HHV6 infections after HSCT. Sci Transl Med (2014) 6:242ra83. doi: 10.1126/scitranslmed.3008825 PMC4181611 24964991

[5] Leen AM Bollard CM Mendizabal AM Shpall EJ Szabolcs P Antin JH. Multicenter study of banked third-party virus-specific T cells to treat severe viral infections after hematopoietic stem cell transplantation. Blood (2013) 121:5113–23. doi: 10.1182/blood-2013-02-486324 PMC3695359 23610374

[6] Nelson AS Heyenbruch D Rubinstein JD Sabulski A Jodele S Thomas S. Virus-specific T-cell therapy to treat BK polyomavirus infection in bone marrow and solid organ transplant recipients. Blood Adv (2020) 4:5745–54. doi: 10.1182/bloodadvances.2020003073 PMC7686882 33216887

[7] Prockop S Doubrovina E Suser S Heller G Barker J Dahi P. Off-the-shelf EBV-specific T cell immunotherapy for rituximab-refractory EBV-associated lymphoma following transplantation. J Clin Invest (2020) 130:733–47. doi: 10.1172/JCI121127 PMC6994129 31689242

[8] Prockop SE Hasan A Doubrovina E Dahi PB Rodriguez-Sanchez I Curry M. Third-party cytomegalovirus-specific T cells improved survival in refractory cytomegalovirus viremia after hematopoietic transplant. J Clin Invest (2023) 133(10):e165476. doi: 10.1172/JCI165476 36951958 PMC10178844

[9] Gerdemann U Keirnan JM Katari UL Yanagisawa R Christin AS Huye LE. Rapidly generated multivirus-specific cytotoxic T lymphocytes for the prophylaxis and treatment of viral infections. Mol Ther (2012) 20:1622–32. doi: 10.1038/mt.2012.130 PMC3412490 22801446

[10] Tzannou I Papadopoulou A Naik S Leung K Martinez CA Ramos CA. Off-the-shelf virus-specific T cells to treat BK virus, human herpesvirus 6, cytomegalovirus, epstein-barr virus, and adenovirus infections after allogeneic hematopoietic stem-cell transplantation. J Clin Oncol (2017) 35:3547–57. doi: 10.1200/JCO.2017.73.0655 PMC5662844 28783452

[11] Grau-Vorster M Lopez-Montanes M Canto E Vives J Oliver-Vila I Barba P. Characterization of a cytomegalovirus-specific T lymphocyte product obtained through a rapid and scalable production process for use in adoptive immunotherapy. Front Immunol (2020) 11:271. doi: 10.3389/fimmu.2020.00271 32161589 PMC7052482

[12] Wonderlich J Shearer G Livingstone A Brooks A Soloski MJ Presby MM. Induction and measurement of cytotoxic T lymphocyte activity. Curr Protoc Immunol (2018) 120:3 11 1–3 11 29. doi: 10.1002/cpim.38 29512145

[13] Koukoulias K Papadopoulou A Kouimtzidis A Papayanni PG Papaloizou A Sotiropoulos D. Non-transplantable cord blood units as a source for adoptive immunotherapy of leukaemia and a paradigm of circular economy in medicine. Br J Haematol (2021) 194:158–67. doi: 10.1111/bjh.17464 34036576

[14] Papayanni PG Chasiotis D Koukoulias K Georgakopoulou A Iatrou A Gavriilaki E. Vaccinated and convalescent donor-derived severe acute respiratory syndrome coronavirus 2-specific T cells as adoptive immunotherapy for high-risk coronavirus disease 2019 patients. Clin Infect Dis (2021) 73:2073–82. doi: 10.1093/cid/ciab371 PMC8135332 33905481

[15] Lyons AB Blake SJ Doherty KV. Flow cytometric analysis of cell division by dilution of CFSE and related dyes. Curr Protoc Cytom Chapter (2013) 9:9 11 1–9 11 12. doi: 10.1002/0471142956.cy0911s64 23546777

[16] Parish CR. Fluorescent dyes for lymphocyte migration and proliferation studies. Immunol Cell Biol (1999) 77:499–508. doi: 10.1046/j.1440-1711.1999.00877.x 10571670

[17] de Wolf C van de Bovenkamp M Hoefnagel M. Regulatory perspective on in *vitro* potency assays for human T cells used in anti-tumor immunotherapy. Cytotherapy (2018) 20:601–22. doi: 10.1016/j.jcyt.2018.01.011 29598903

[18] Shresta S Pham CT Thomas DA Graubert TA Ley TJ. How do cytotoxic lymphocytes kill their targets? Curr Opin Immunol (1998) 10:581–7. doi: 10.1016/S0952-7915(98)80227-6 9794837

[19] Jedema I van der Werff NM Barge RM Willemze R Falkenburg JH. New CFSE-based assay to determine susceptibility to lysis by cytotoxic T cells of leukemic precursor cells within a heterogeneous target cell population. Blood (2004) 103:2677–82. doi: 10.1182/blood-2003-06-2070 14630824

[20] Dicker F Kater AP Fukuda T Kipps TJ. Fas-ligand (CD178) and TRAIL synergistically induce apoptosis of CD40-activated chronic lymphocytic leukemia B cells. Blood (2005) 105:3193–8. doi: 10.1182/blood-2003-10-3684 15339846

[21] Hildemann SK Eberlein J Davenport B Nguyen TT Victorino F Homann D. High efficiency of antiviral CD4(+) killer T cells. PloS One (2013) 8:e60420. doi: 10.1371/journal.pone.0060420 23565245 PMC3614903

[22] Jellison ER Kim SK Welsh RM. Cutting edge: MHC class II-restricted killing in *vivo* during viral infection. J Immunol (2005) 174:614–8. doi: 10.4049/jimmunol.174.2.614 15634878

[23] Martorelli D Muraro E Merlo A Turrini R Rosato A Dolcetti R. Role of CD4+ cytotoxic T lymphocytes in the control of viral diseases and cancer. Int Rev Immunol (2010) 29:371–402. doi: 10.3109/08830185.2010.489658 20635880

[24] Porakishvili N Kardava L Jewell AP Yong K Glennie MJ Akbar A. Cytotoxic CD4+ T cells in patients with B cell chronic lymphocytic leukemia kill *via* a perforin-mediated pathway. Haematologica (2004) 89:435–43. 15075077

[25] Williams NS Engelhard VH. Identification of a population of CD4+ CTL that utilizes a perforin- rather than a Fas ligand-dependent cytotoxic mechanism. J Immunol (1996) 156:153–9. doi: 10.4049/jimmunol.156.1.153 8598456

[26] Chen K Chen L Zhao P Marrero L Keoshkerian E Ramsay A. FL-CTL assay: fluorolysometric determination of cell-mediated cytotoxicity using green fluorescent protein and red fluorescent protein expressing target cells. J Immunol Methods (2005) 300:100–14. doi: 10.1016/j.jim.2005.02.010 15899496

[27] Godoy-Ramirez K Makitalo B Thorstensson R Sandstrom E Biberfeld G Gaines H. A novel assay for assessment of HIV-specific cytotoxicity by multiparameter flow cytometry. Cytometry A (2005) 68:71–80. doi: 10.1002/cyto.a.20189 16228974

[28] Kim GG Donnenberg VS Donnenberg AD Gooding W Whiteside TL. A novel multiparametric flow cytometry-based cytotoxicity assay simultaneously immunophenotypes effector cells: comparisons to a 4 h 51Cr-release assay. J Immunol Methods (2007) 325:51–66. doi: 10.1016/j.jim.2007.05.013 17617419 PMC2040258

[29] Lecoeur H Fevrier M Garcia S Riviere Y Gougeon ML. A novel flow cytometric assay for quantitation and multiparametric characterization of cell-mediated cytotoxicity. J Immunol Methods (2001) 253:177–87. doi: 10.1016/S0022-1759(01)00359-3 11384679

[30] Liu L Chahroudi A Silvestri G Wernett ME Kaiser WJ Safrit JT. Visualization and quantification of T cell-mediated cytotoxicity using cell-permeable fluorogenic caspase substrates. Nat Med (2002) 8:185–9. doi: 10.1038/nm0202-185 11821904

[31] Lin L Couturier J Yu X Medina MA Kozinetz CA Lewis DE. Granzyme B secretion by human memory CD4 T cells is less strictly regulated compared to memory CD8 T cells. BMC Immunol (2014) 15:36. doi: 10.1186/s12865-014-0036-1 25245659 PMC4195902

[32] Shafer-Weaver K Sayers T Strobl S Derby E Ulderich T Baseler M. The Granzyme B ELISPOT assay: an alternative to the 51Cr-release assay for monitoring cell-mediated cytotoxicity. J Transl Med (2003) 1:14. doi: 10.1186/1479-5876-1-14 14697097 PMC317386

[33] Borst J Ahrends T Babala N Melief CJM Kastenmuller W. CD4(+) T cell help in cancer immunology and immunotherapy. Nat Rev Immunol (2018) 18:635–47. doi: 10.1038/s41577-018-0044-0 30057419

[34] Swain SL McKinstry KK Strutt TM. Expanding roles for CD4(+) T cells in immunity to viruses. Nat Rev Immunol (2012) 12:136–48. doi: 10.1038/nri3152 PMC3764486 22266691

[35] Cenerenti M Saillard M Romero P Jandus C. The era of cytotoxic CD4 T cells. Front Immunol (2022) 13:867189. doi: 10.3389/fimmu.2022.867189 35572552 PMC9094409

[36] Kravtsov DS Erbe AK Sondel PM Rakhmilevich AL. Roles of CD4+ T cells as mediators of antitumor immunity. Front Immunol (2022) 13:972021. doi: 10.3389/fimmu.2022.972021 36159781 PMC9500154

[37] Oh DY Fong L. Cytotoxic CD4(+) T cells in cancer: Expanding the immune effector toolbox. Immunity (2021) 54:2701–11. doi: 10.1016/j.immuni.2021.11.015 PMC8809482 34910940

[38] Gamadia LE Remmerswaal EB Weel JF Bemelman F van Lier RA Ten Berge IJ. Primary immune responses to human CMV: a critical role for IFN-gamma-producing CD4+ T cells in protection against CMV disease. Blood (2003) 101:2686–92. doi: 10.1182/blood-2002-08-2502 12411292

[39] van Leeuwen EM Remmerswaal EB Heemskerk MH ten Berge IJ van Lier RA. Strong selection of virus-specific cytotoxic CD4+ T-cell clones during primary human cytomegalovirus infection. Blood (2006) 108:3121–7. doi: 10.1182/blood-2006-03-006809 16840731

[40] van Leeuwen EM Remmerswaal EB Vossen MT Rowshani AT Wertheim-van Dillen PM van Lier RA. Emergence of a CD4+CD28- granzyme B+, cytomegalovirus-specific T cell subset after recovery of primary cytomegalovirus infection. J Immunol (2004) 173:1834–41. doi: 10.4049/jimmunol.173.3.1834 15265915

[41] Wilkinson TM Li CK Chui CS Huang AK Perkins M Liebner JC. Preexisting influenza-specific CD4+ T cells correlate with disease protection against influenza challenge in humans. Nat Med (2012) 18:274–80. doi: 10.1038/nm.2612 22286307

[42] Smyk-Pearson S Tester IA Klarquist J Palmer BE Pawlotsky JM Golden-Mason L. Spontaneous recovery in acute human hepatitis C virus infection: functional T-cell thresholds and relative importance of CD4 help. J Virol (2008) 82:1827–37. doi: 10.1128/JVI.01581-07 PMC2258714 18045940

[43] Ma Y Yuan B Zhuang R Zhang Y Liu B Zhang C. Hantaan virus infection induces both Th1 and ThGranzyme B+ cell immune responses that associated with viral control and clinical outcome in humans. PloS Pathog (2015) 11:e1004788. doi: 10.1371/journal.ppat.1004788 25836633 PMC4383613

[44] Soghoian DZ Jessen H Flanders M Sierra-Davidson K Cutler S Pertel T. HIV-specific cytolytic CD4 T cell responses during acute HIV infection predict disease outcome. Sci Transl Med (2012) 4:123ra25. doi: 10.1126/scitranslmed.3003165 PMC3918726 22378925

[45] Rosenberg ES Billingsley JM Caliendo AM Boswell SL Sax PE Kalams SA. Vigorous HIV-1-specific CD4+ T cell responses associated with control of viremia. Science (1997) 278:1447–50. doi: 10.1126/science.278.5342.1447 9367954

[46] Alspach E Lussier DM Miceli AP Kizhvatov I DuPage M Luoma AM. MHC-II neoantigens shape tumour immunity and response to immunotherapy. Nature (2019) 574:696–701. doi: 10.1038/s41586-019-1671-8 31645760 PMC6858572

[47] Hunder NN Wallen H Cao J Hendricks DW Reilly JZ Rodmyre R. Treatment of metastatic melanoma with autologous CD4+ T cells against NY-ESO-1. N Engl J Med (2008) 358:2698–703. doi: 10.1056/NEJMoa0800251 PMC3277288 18565862

[48] Johnson DB Estrada MV Salgado R Sanchez V Doxie DB Opalenik SR. Melanoma-specific MHC-II expression represents a tumour-autonomous phenotype and predicts response to anti-PD-1/PD-L1 therapy. Nat Commun (2016) 7:10582. doi: 10.1038/ncomms10582 26822383 PMC4740184

[49] Rodig SJ Gusenleitner D Jackson DG Gjini E Giobbie-Hurder A Jin C. MHC proteins confer differential sensitivity to CTLA-4 and PD-1 blockade in untreated metastatic melanoma. Sci Transl Med (2018) 10(450):eaar3342. doi: 10.1126/scitranslmed.aar3342 30021886

[50] Tang XX Shimada H Ikegaki N. Clinical relevance of CD4 cytotoxic T cells in high-risk neuroblastoma. Front Immunol (2021) 12:650427. doi: 10.3389/fimmu.2021.650427 33968044 PMC8101497

[51] Tran E Turcotte S Gros A Robbins PF Lu YC Dudley ME. Cancer immunotherapy based on mutation-specific CD4+ T cells in a patient with epithelial cancer. Science (2014) 344:641–5. doi: 10.1126/science.1251102 PMC6686185 24812403

[52] Zhang X Gao L Meng K Han C Li Q Feng Z. Characterization of CD4(+) T cell-mediated cytotoxicity in patients with multiple myeloma. Cell Immunol (2018) 327:62–7. doi: 10.1016/j.cellimm.2018.02.009 29454645

[53] Oh DY Kwek SS Raju SS Li T McCarthy E Chow E. Intratumoral CD4(+) T cells mediate anti-tumor cytotoxicity in human bladder cancer. Cell (2020) 181:1612–1625 e13. doi: 10.1016/j.cell.2020.05.017 32497499 PMC7321885

[54] Cachot A Bilous M Liu YC Li X Saillard M Cenerenti M. Tumor-specific cytolytic CD4 T cells mediate immunity against human cancer. Sci Adv (2021) 7(9):eabe3348. doi: 10.1126/sciadv.abe3348 33637530 PMC7909889

