# Supplementary material for: Assessment of the cytolytic potential of a multivirus-targeted T cell therapy using a vital dye-based, flow cytometric assay
Source: Front Immunol. 2023 Dec 22;14:1299512. doi: 10.3389/fimmu.2023.1299512 (PMC10766817; doi:10.3389/fimmu.2023.1299512)
Supplement: Supplementary file 1 [file DataSheet_1.docx]

**Supplementary materials**

**Supplementary figure legends**

**Suppl. Figure 1. Surface phenotype of multiVSTs as assessed by flow cytometry**. Summary data from n=5 multiVST lines are shown as mean ± SEM.

**Suppl. Figure 2. In vitro functional profiling of multiVSTs.** A: IFNγ production by multiVSTs as assessed by ELISpot assay using pooled pepmixes of each individual target virus as a stimulus. Results are expressed as SFC/2x10^5^ input cells; a threshold of 50 SFC/2x10^5^ was set to define specificity. Unstimulated control values have been subtracted. B: Granzyme B production by multiVSTs as assessed by ELISpot assay using pooled pepmixes of each individual target virus as a stimulus. Results are expressed as SFC/2x10^5^ input cells. Unstimulated control values have been subtracted. C: Upregulation of CD107a by multiVSTs following stimulation by individual target viruses. Unstimulated control values have been subtracted. D: Specific cytotoxic activity of multiVSTs against viral antigen-loaded autologous PHA blasts as evaluated in a standard 5-hour ^51^Cr release assay at 40:1 E:T ratio. The threshold for specific lytic activity against each individual target virus (indicated by the dashed line) was set at 10%. Four individual donor examples are shown, along with summary data depicted as mean ± SEM (far right column). SFC: spot forming cells.

**Suppl. Figure 3. Optimization of the CFSE platform for assessing cytotoxicity of multiVSTs.** A: Assessment of the lytic capacity of multiVSTs against autologous PHA blasts loaded with a mastermix of target viruses (AdV, BKV, CMV, EBV, HHV6) using either a standard 5-hour ^51^Cr release assay (grey bar) or a 5-hour CFSE-based assay (pink bar). Results are shown as mean ± SD (n=2). B: Impact of co-culture duration (5 hours vs 16 hours) on detection of multiVST-mediated killing of mastermix-pulsed autologous PHA blasts using the CFSE platform (mean± SEM; n=6). Cytotoxicity was evaluated at 40:1 E:T. Differences between data sets were analyzed using a 2-tailed Student’s t-test. P-values <0.05 were considered significant. ns: non-significant; **: p=0.003

**Suppl. Figure 4: The CFSE-based assay is superior to standard ^51^Cr release in the detection of specific cytolytic activity of multiVSTs.** Assessment of the lytic capacity of multiVSTs against autologous PHA blasts loaded with individual target viruses using either a standard 5-hour ^51^Cr release assay (grey bars) or the 16-hour CFSE-based assay (red bars). Cytotoxicity was evaluated at 40:1 E:T following a 1-hour target antigen-pulsing. Dashed lines represent the threshold for specific lytic activity (>10%). Diamonds indicate antiviral specificity of multiVSTs as assessed by IFNγ ELISpot (dark blue: >50 SFC/2x10^5^; light blue: <50 SFC/2x10^5^; specificity threshold was set at 50 SFC). Seven individual donor examples are shown.

**Suppl. Figure 5: The CFSE-based assay allows for successful detection of CD4 T cell-mediated specific killing.** Purity of the magnetically sorted CD4+ VST fraction as assessed by flow cytometry (left column) and measurement of the lytic capacity of CD4+ multiVSTs against autologous PHA blasts loaded with individual target viruses using either a standard 5-hour ^51^Cr release assay (grey bars – middle column) or the 16-hour CFSE-based assay (red bars – right column). Cytotoxicity was evaluated at 40:1 E:T following a 1-hour target antigen-pulsing. Three individual donor examples (panels A, B and C) are shown. Dashed lines represent the threshold for specific lytic activity (>10%).

**Suppl. Figure 6: The CFSE-based assay is robust and compatible with high-throughput flow cytometric platforms.** A: Compatibility of the CFSE-based assay with different instrumentation (left side – Gallios, right side – CytoFlex); a representative donor is shown. B: MultiVST-mediated specific killing of target cells was reproducibly assessed using either instrument (n=3; mean± SD). Autologous PHA blasts loaded with a mastermix of target viruses (AdV, BKV, CMV, EBV, HHV6) served as cognate antigen-pulsed targets. Cytotoxicity was evaluated at 40:1 E:T following a 1-hour target antigen-pulsing and a 16-hour E:T co-culture. Differences between data sets were analyzed using a 2-tailed Student’s t-test. P-values <0.05 were considered significant. ns: non-significant.

**Suppl. Figure 1**


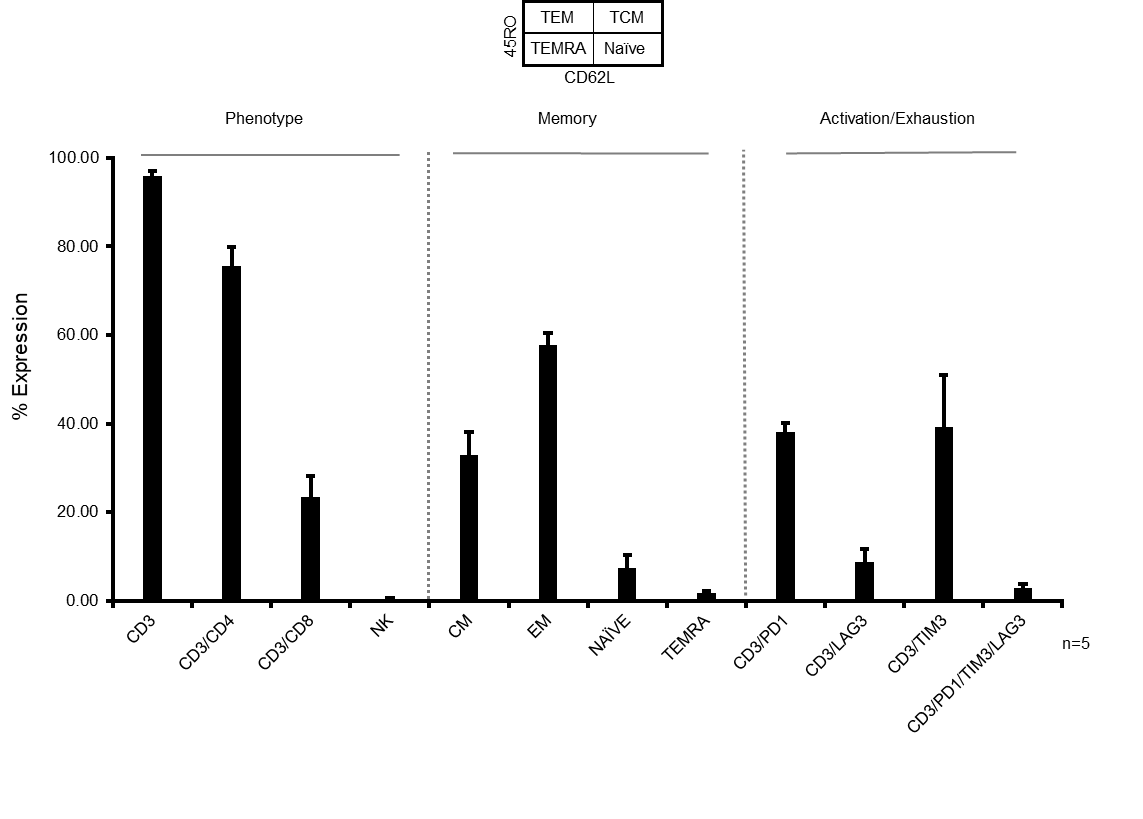


**Suppl. Figure 2**

**
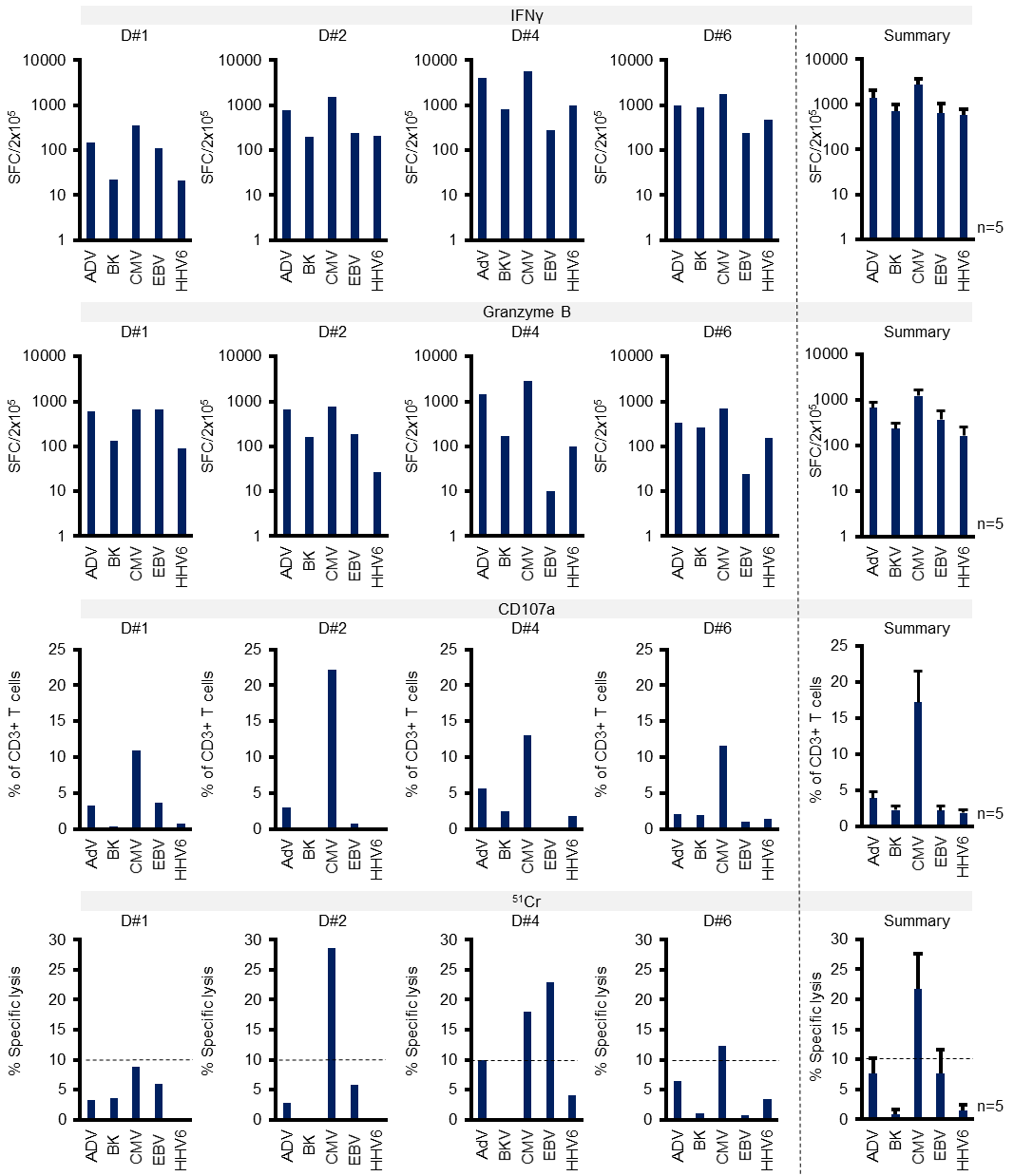
**

D

C

A

B

**Suppl. Figure 3**

**
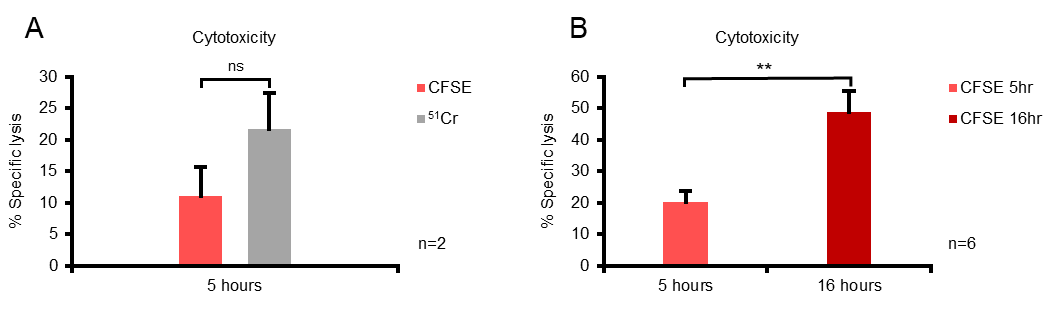
**

**Suppl. Figure 4**

**
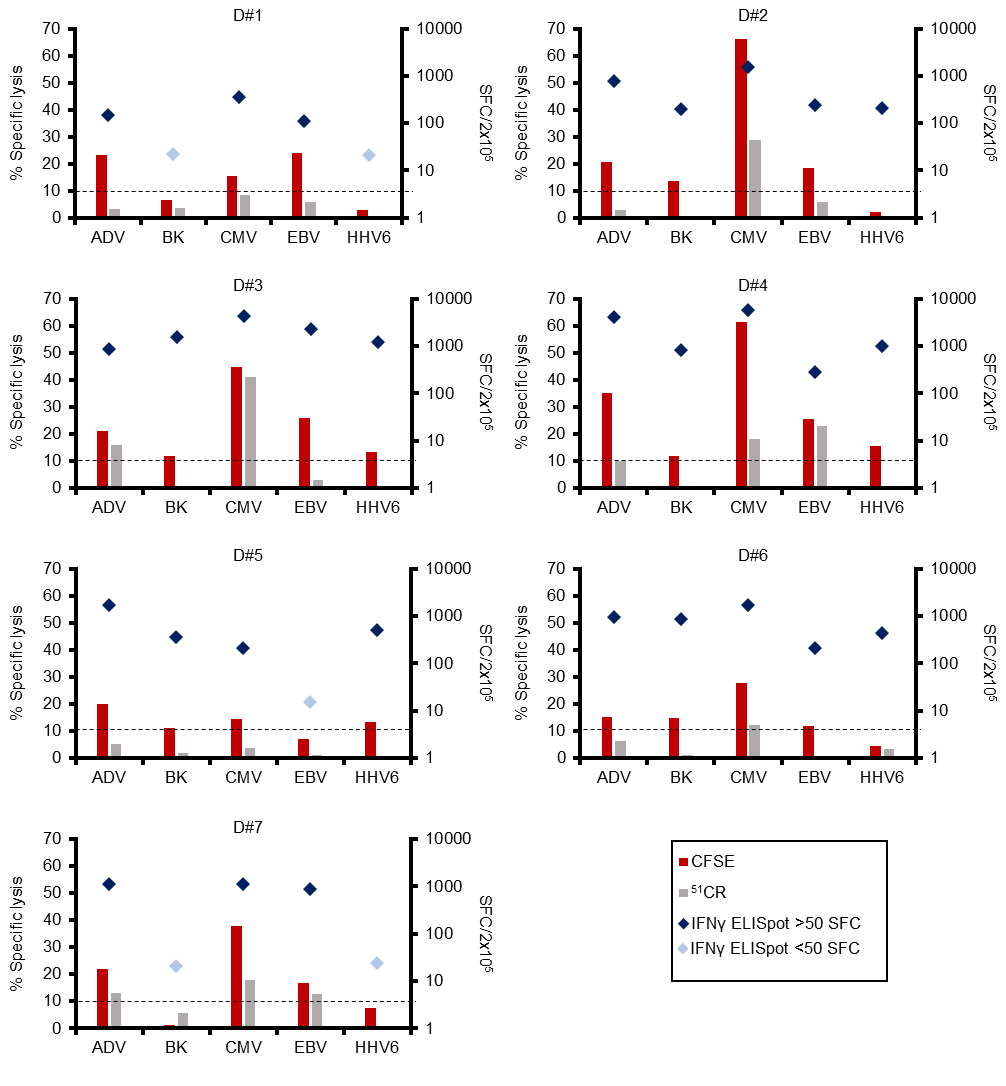
**

**Suppl. Figure 5**

**
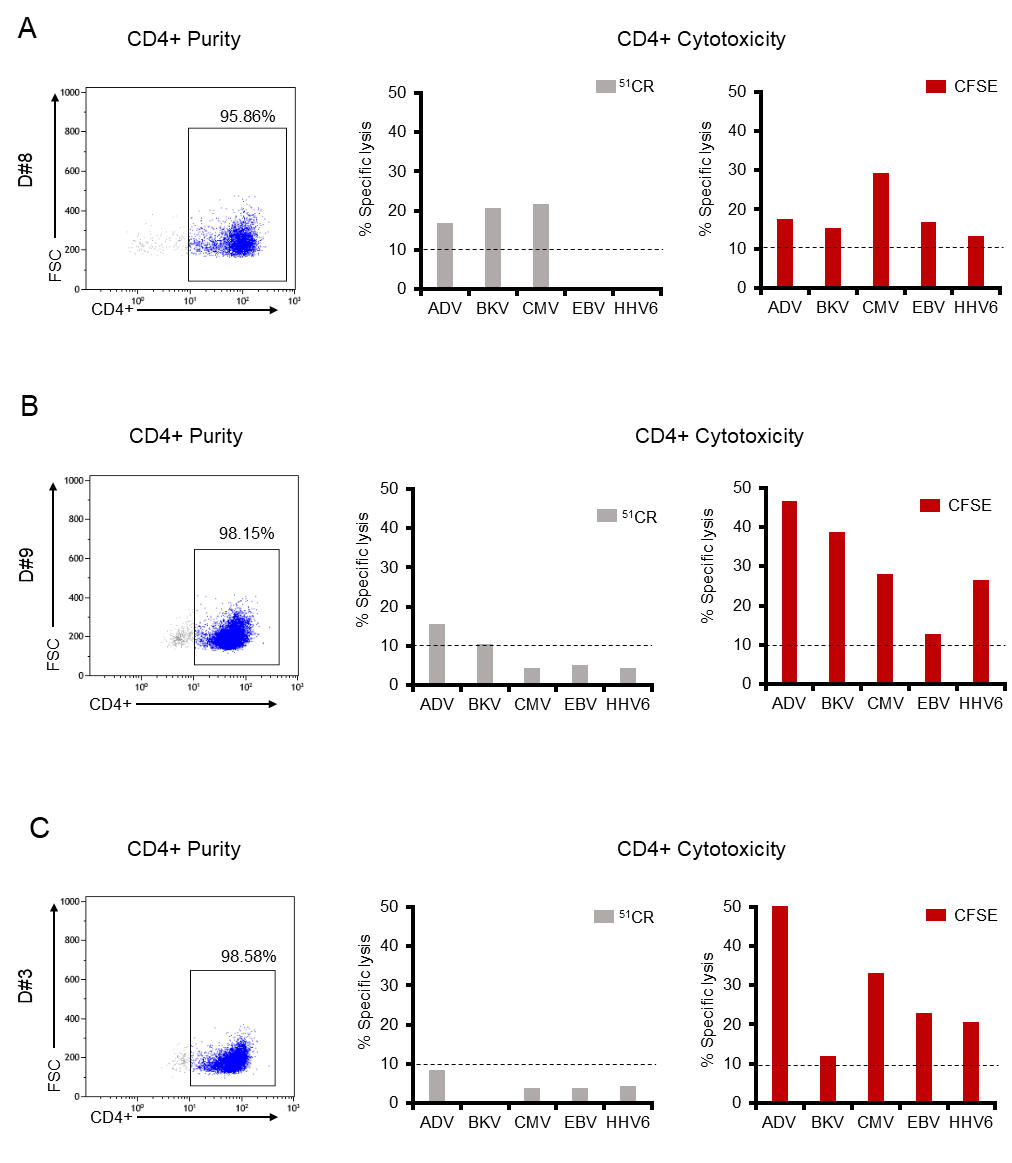
**

**Suppl. Figure 6**

**
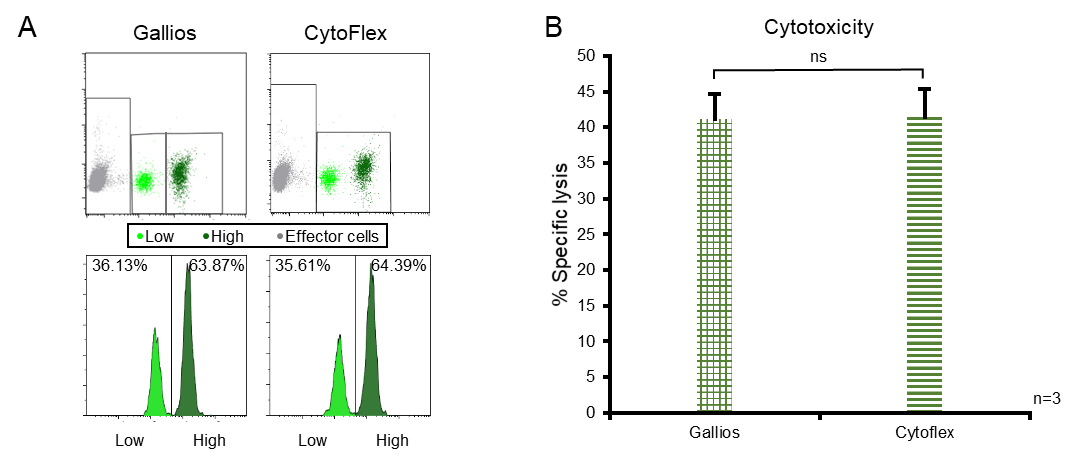
**
